# A New Method to Estimate Changes in Glacier Surface Elevation Based on Polynomial Fitting of Sparse ICESat—GLAS Footprints

**DOI:** 10.3390/s17081803

**Published:** 2017-08-05

**Authors:** Tianjin Huang, Li Jia, Massimo Menenti, Jing Lu, Jie Zhou, Guangcheng Hu

**Affiliations:** 1 State Key Laboratory of Remote Sensing Science, Institute of Remote Sensing and Digital Earth, Chinese Academy of Sciences, Beijing 100101, China; hangtj@radi.ac.cn (T.H.); M.Menenti@tudelft.nl (M.M.); lujing@radi.ac.cn (J.L.); zhoujie@radi.ac.cn (J.Z.); hugc@radi.ac.cn (G.H.); 2 Joint Center for Global Change Studies, Beijing 100875, China; 3 University of Chinese Academy of Sciences, Beijing 100049, China; 4 Department of Geoscience and Remote Sensing, Delft University of Technology, Delft 2600, The Netherlands

**Keywords:** glacier thickness change, ICESat, polynomial fitting method

## Abstract

We present in this paper a polynomial fitting method applicable to segments of footprints measured by the Geoscience Laser Altimeter System (GLAS) to estimate glacier thickness change. Our modification makes the method applicable to complex topography, such as a large mountain glacier. After a full analysis of the planar fitting method to characterize errors of estimates due to complex topography, we developed an improved fitting method by adjusting a binary polynomial surface to local topography. The improved method and the planar fitting method were tested on the accumulation areas of the Naimona’nyi glacier and Yanong glacier on along-track facets with lengths of 1000 m, 1500 m, 2000 m, and 2500 m, respectively. The results show that the improved method gives more reliable estimates of changes in elevation than planar fitting. The improved method was also tested on Guliya glacier with a large and relatively flat area and the Chasku Muba glacier with very complex topography. The results in these test sites demonstrate that the improved method can give estimates of glacier thickness change on glaciers with a large area and a complex topography. Additionally, the improved method based on GLAS Data and Shuttle Radar Topography Mission-Digital Elevation Model (SRTM-DEM) can give estimates of glacier thickness change from 2000 to 2008/2009, since it takes the 2000 SRTM-DEM as a reference, which is a longer period than 2004 to 2008/2009, when using the GLAS data only and the planar fitting method.

## 1. Introduction

Glacier thickness change is one of the most important observations to estimate glacier mass balance. According to the Fifth Assessment Report of the Intergovernmental Panel on Climate Change (IPCC AR5) [1], the melting of mountain glaciers and polar ice caps has made large contributions to the rise of sea level. In previous studies, several methods have been applied to measure glacier thickness change: (1) in situ measurements; (2) difference of multi-temporal Digital Elevation Models (DEM) [2,3,4]; and (3) satellite radar altimetry [5]. Direct measurements mainly use GPS (Global Positioning System) or stakes to monitor glacier surface elevation changes, which is labor- and time-consuming. Differencing multi-temporal DEMs [3,4] have the advantage of capturing the spatial distribution of glacier thickness change. The challenge is to generate accurate DEMs. Remote sensing methods of deriving DEMs include photogrammetry techniques and interferometric synthetic aperture radar (InSAR) [6], both of which are time-consuming and complicated because of the lack of contrast and temporal de-coherence [7,8]. Satellite radar altimetry is another method to measure glacier thickness change. Accurate elevation data were acquired during 2003–2009 by the Geoscience Laser Altimeter System (GLAS) [5,9,10,11]. Two types of glacier thickness change measurements can be achieved using GLAS data: (a) differences in time at cross-over points; and (b) sampling of the same ground-track over time. The problem with method (a) is that cross-over points of ascending and descending tracks are few in regions apart from Greenland and Antarctica [5]. Method (b) makes better use of the dense, along-track sampling by GLAS, but is influenced by topographic variation due to the non-overlapping repeat of actual ground tracks. Three algorithms have been used to account for across-track topographic variations and estimate glacier thickness change: (1) combining areal samples over time within a limited glacier facet using an external DEM [5]; (2) the planar fitting method to fit a plane to areal samples taken at different times within a limited glacier facet [5]; and (3) estimating a temporal trend by differencing a reference DEM [10]. Moholdt [5] argued that algorithm (2) performs better after comparing algorithms (1) and (2). However, algorithm (2) may have a bias when applied to glaciers with complex surface topography, such as large mountain glaciers or large areas in polar regions. 

The main idea of the planar fitting method is that the difference in glacier surface modelled (locally) as a plane on the basis of observed footprints gives an estimates of glacier thickness change. It can separate temporal elevation changes from topographic variations by fitting a plane to segments of repeat-tracks of GLAS data within a facet of the surface using least-squares regression technique [2,12]. The width of the facet is usually determined by the maximum distance spanned by the footprints over the period of observation. In theory, the method can give a result without bias when two assumptions are satisfied:

(1) The glacier surface can be fitted by plane; and
(2) The glacier thickness change within a planar facet is constant and the shape of glacier surface remains unchanged with time, even though it moves vertically.

Both Assumptions 1 and 2 are likely to hold locally, i.e., when a small facet of the glacier surface is considered. Nevertheless, given the large across-track spacing of GLAS footprints and very complex glacier surface in the mid-low-latitude mountain glaciers, Assumptions 1 and 2 will not hold for all glacier facets. Thus, in this paper, we will analyze the error sources of the planar fitting method when applying it to large mountain glaciers. According to the results of the analysis, we will improve the planar method to reduce the bias caused by complex topography. A new approach will be presented to combine GLAS data and the Shuttle Radar Topography Mission (SRTM) DEM. Finally, the improved method will be applied to the test sites on four glaciers in the Tibetan Plateau in China to test its performance in comparison with the planar method.

## 2. Data and Experiment Sites

### 2.1. ICESat/GLAS Altimetry Data

As part of National Aeronautics and Space Administration (NASA)’s Earth Observing System, the Geoscience Laser Altimeter System (GLAS) was a space-based LIDAR sensor on board the Ice Cloud and Elevation Satellite (ICESat), and was launched on 13 January 2003. It was operated in a 91-day exact repeat orbit with a 33-day sub-cycle [13] and obtained along-track measurements with a ~170 m distance between subsequent footprints. As ICESat precision spacecraft pointing control was not used in mid-low latitudes, individual repeated tracks do not match exactly, but can be separated across track by several hundred meters to several kilometers. Each footprint is nearly a circle on flat terrain with around 66 m diameter. GLAS data have been proved to have a vertical accuracy of about 10 cm over flat terrain and horizontal accuracy of about 5 m [14,15,16]. In this study, GLAS Global Land Surface Altimetry Data (GLA14) was exploited, which provides surface elevations over land. It provided data in three seasons, i.e., winter (February/March), summer (May/June), and autumn (October/November) from 2003 to 2009. Only single-season data were used to avoid the effect of glacier elevation changes between seasons, such as due to snowfall and melting [10]. Only GLAS data in February/March were used in combination with the SRTM DEM which was acquired in February 2000. For the representativeness of the estimated changes, at least four years of GLAS data were required.

### 2.2. SRTM-DEM Data

**Figure 1:**
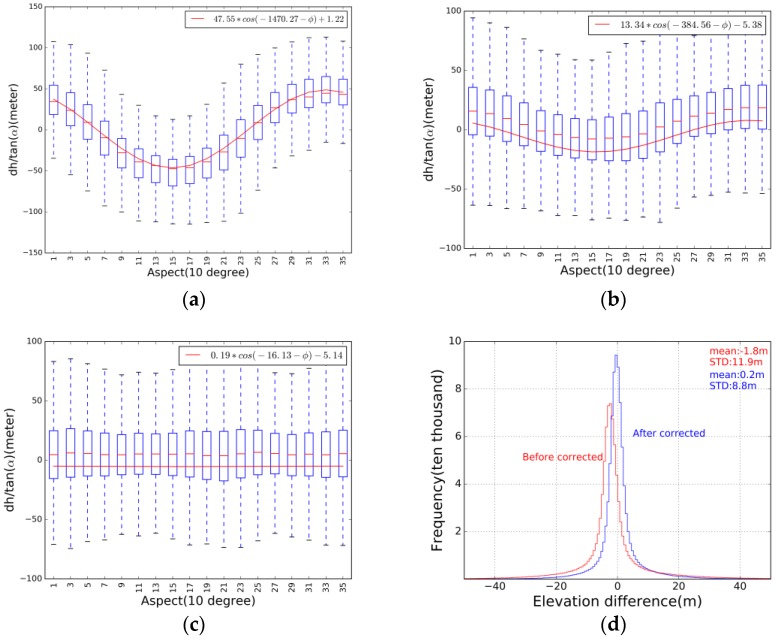
(**a**) The relationship between aspect and the elevation difference of GLAS data and SRTM DEM before universal co-registration correction; (**b**) the relationship between the aspect and the elevation difference of GLAS and SRTM after the first universal co-registration; (**c**) The relationship between the aspect and the elevation difference of GLAS and SRTM after the final universal co-registration; and (**d**) the histogram of the elevation differences before and after applying the universal co-registration.

The SRTM, flown on board the space shuttle Endeavour 11–22 February 2000, measured near-global elevations using single-pass synthetic aperture radar (SAR) interferometry [17]. In this study SRTM void-filled data was used [18]. This DEM data has a resolution of 90 m corresponding to 3-arc seconds at the equator [19]. Some studies indicated that SRTM elevations may have horizontal shifts due to post-processing, such as vertical merging of overlapping elevation measurements and horizontal mosaicking [17]. Thus, a universal co-registration correction was applied to SRTM DEM and GLAS data according to the relationship between elevation difference and aspect [17,20,21]. GLAS and SRTM data for bare regions only were used in this universal co-registration correction. Figure 1a,b show the obvious cosine relationship between GLAS data and SRTM DEM before universal co-registration and after first universal co-registration. As it is difficult to remove the shift after a universal co-registration, this process must be iterated [17]. After iterative computations, a 64.7 m shift to the northwest by 28.2° between SRTM DEM and GLAS footprints was found. After co-registration, the mean elevation difference improved from −1.8 m to 0.2 m. The standard error of the estimated elevation also decreased from 11.9 m to 8.8 m.

### 2.3. Experiment Sites

**Figure 2:**
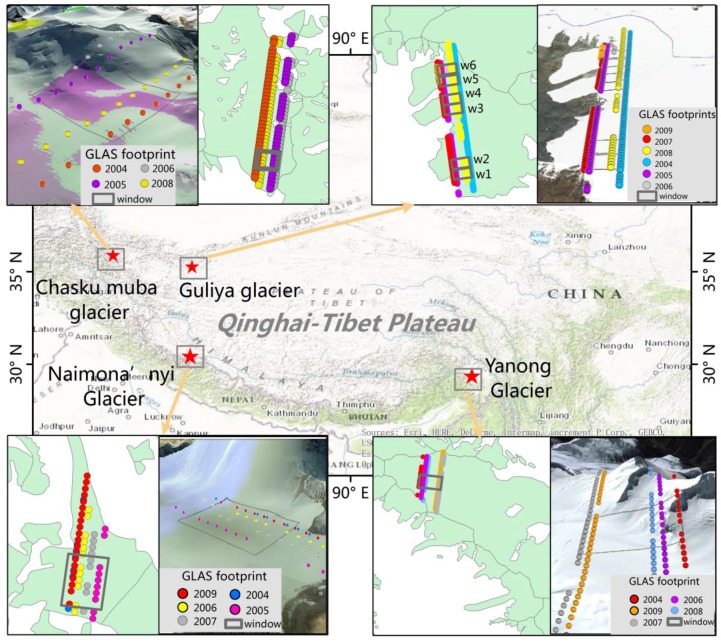
Map of four test sites on Yanong, Naimona’nyi, Guliya, and Chasku Muba glaciers, which are located in Nyainqentanglha Mountains, Himalayan Mountains, Kunlun Mountains, and Karakoram Mountains, respectively; each image (Google Earth) shows the real glacial surface of each test site. Dots represent the position of actual GLAS footprints and each track represents one year of GLAS data. The color of footprints indicates the year of acquisition. Five-year GLAS data, five-year GLAS data, four-year GLAS data, and six-year GLAS data were acquired in the Naimona’nyi Glacier, the Yanong Glacier, the Chasku Muba glacier, and the Guliya glacier, respectively. Grey contour lines (rectangular window) indicate the along-track facet applied in the evaluation. The width of the facet is determined by the available footprints across the tracks acquired between 2003 and 2009. The length of the along-track window is 1 km.

Four experimental sites were chosen (Figure 2): Yanong glacier (96.56° *E*, 29.38° *N*) with an area of 17.9 km^2^, which is located southeast of the Nyainqentanglha Range; Naimona’nyi glacier (81.32° *E*, 30.45° *N*) with an area of 7.3 km^2^, which is located to the west of the Himalayas; Guliya glacier (81.47° *E*, 35.24° *N*) with an area of 111.3 km^2^, which is located in the Kunlun Mountains; and Chasku Muba glacier (77.16° *E*, 35.90° *N*) with an area of 43.7 km^2^, which is located in the Karakoram Mountains. The average roughness that mirrors the complexity of the glacier surfaces were calculated for each experiment based on SRTM-DEM. The roughness here is defined as the root-mean-square of the differences between SRTM-DEM and the locally best-fitting plane based on SRTM-DEM [22]. The size of local plane here is equal to the size of the rectangular as shown in Figure 2. The results showed that Naimona’nyi and Guliya glaciers have higher roughness, and Yanong and Chasku Muba glaciers have lower roughness (Table 1).

### 2.4. Experiments and Data Preparation

Naimona’nyi and Yanong glaciers were used to test the influence of the window length shown in Figure 2 on the result calculated by the improved method and the planar fitting method. Chasku Muba and Yanong glacier surfaces have very complex topography, which were used to test the performance of the improved method. Guliya glacier was used to show that the improved method could be applied to a large glacier. 

Three steps of pre-processing are needed before using the ICESat/GLAS data: (1) convert ICESat/GLAS ellipsoid (TOPEX/Poseidon) to a WGS84 ellipsoid; (2) convert the geoid to Earth Gravitational Model 1996 (EGM96); and (3) remove abnormal values affected by detector saturation. We selected those footprints whose return echoes are not, or are only slightly, saturated, i.e., when the value of satCorrFlg in the data quality layer is 0 or 1 [23]. In addition, as the ICESat footprints affected by clouds give anomalous elevations, we removed those footprints for which the difference between ICESat elevation and the Shuttle Radar Topography Mission (SRTM) DEM was larger than 100 m [19]. Finally, GLAS data acquired in winter and summer were removed and there were five GLAS ground tracks on the Naimona’nyi Glacier and the Yanong glacier, six GLAS tracks on the Guliya glacier, and four GLAS tracks on the Chasku Muba glacier available for our evaluations. Table 1 shows the number of footprints acquired on each test site.

## 3. Methods

### 3.1. Error Analysis of the Planar Fitting Method

**Figure 3:**
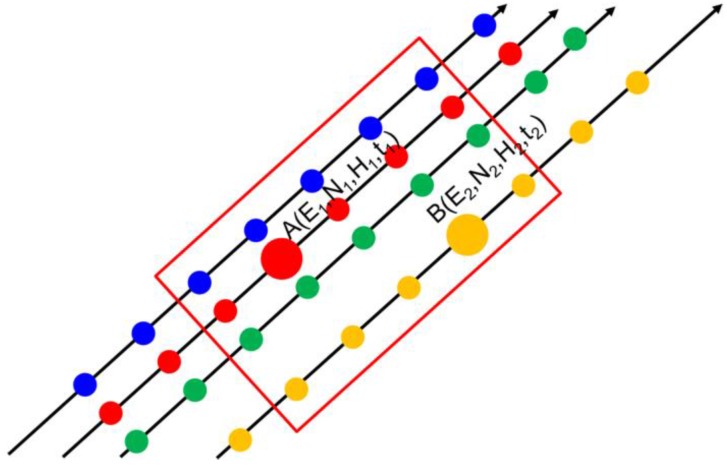
Sketch map of the planes fitted to ground tracks of GLAS. Different colors of footprints denote different tracks. The points *A* and *B* are enlarged footprints characterized by their coordinates (*E*, *N*, *H*) and acquisition time (*t*). The red window shows the sampled facet.

The planar-facets assumption in the planar fitting method is not applicable to complex terrain, such as the mountain glaciers in the Tibetan Plateau, especially when sparse GLAS footprints sample relatively large facets, which need to be further analyzed. To quantify the error source of the planar fitting method, the mathematical derivation of the method is presented here. As illustrated in Figure 3, (*E*, *N*) are the coordinates in the projection coordinate system and *H* is the elevation. We assume that points *A* and *B* are two footprints acquired at locations (*E*_1_, *N*_1_) and (*E*_2_, *N*_2_) on the glacier surface in year *t*_1_ and *t*_2_, respectively, and that the glacial surface is flat and the shape of the facet (red window in Figure 3) does not change. *H*_1_ and *H*_2_ are the elevations measured by GLAS. Therefore, the relationships between points *A*, *B* can be represented by a bilinear equation (plane):

(1) $$H_{1}=\alpha \times E_{1}+\beta \times N_{1}+h_{1}$$

(2) $$H_{2}=\alpha \times E_{2}+\beta \times N_{2}+h_{2}$$

where α, β are the coefficients of the plane equation; $h_{1}$ and $h_{2}$ are constants on the two planes at times *t*_1_ and *t*_2_. The difference between the two plane equations is:

(3) $$dH_{1}=\alpha \times dE_{1}+\beta \times dN_{1}+dh_{1}$$

where $dH_{1}$, $dE_{1}$, $dN_{1}$, and $dh_{1}$ are the differences in (*H*_1_, *H*_2_), (*E*_1_, *E*_2_), (*N*_1_, *N*_2_), and (*h*_1_, *h*_2_), respectively. $dh_{1}$ is the elevation change between glacier surfaces at $t_{1}$ and $t_{2}$, which can be represented as Equation (4):

(4) $$dh_{1}=\left(\frac{dh}{dt}\right)\times \Delta t$$

where $\left(\frac{dh}{dt}\right)$ is the glacier thickness change (m/year); Δt is the difference between $t_{1}$ and $t_{2}.$ Since one such equation can be written for each pair of GLAS point data, when multiple pairs of footprints are available, the differential equations for all pairs of GLAS footprints lead to a system of equations:

(5) $$\left(\begin{array}{c} dH_{1}\\ \vdots \\ dH_{n} \end{array}\right)=\left(\begin{array}{c} dE_{1}\\ \vdots \\ dE_{n} \end{array}\;\begin{array}{c} dN_{1}\\ \vdots \\ dN_{n} \end{array}\;\begin{array}{c} dt_{1}\\ \vdots \\ dt_{n} \end{array}\right)\cdot \left(\begin{array}{c} \alpha \\ \beta \\ \frac{dh}{dt} \end{array}\right)$$

where α, β and $\frac{dh}{dt}$ are unknowns, subscript *n* is the number of differential equations, i.e., of footprint pairs. The least-square regression technique is used to solve the system of equations and to obtain α, β and $\frac{dh}{dt}$.

**Figure 4:**
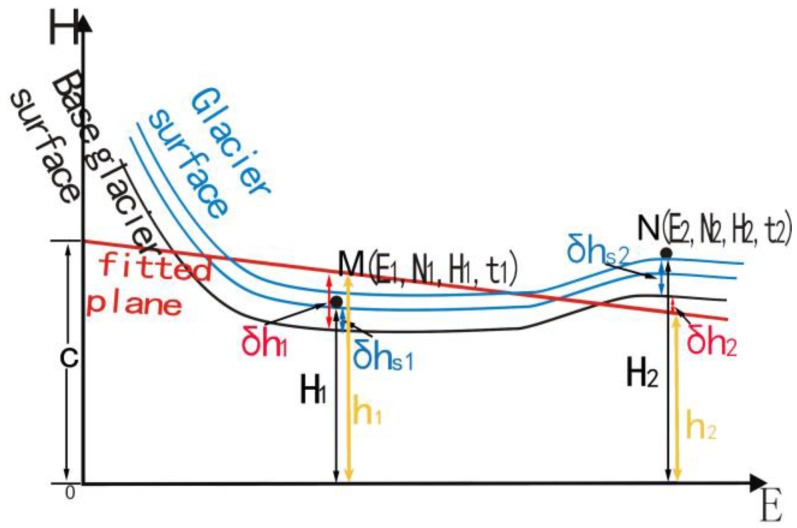
Sketch map of a complex terrain vertical section. The reference glacier surface is the glacier surface in an earlier year (2000 SRTM DEM in this study). $\delta h_{s1}$ and $\delta h_{s2}$ are calculated from the true glacier surface elevation, subtracting the reference glacier surface elevation at locations *M* and *N*. $\delta h_{1}$ and $\delta h_{2}$ are calculated from the reference glacier surface elevation by subtracting the fitted plane elevation at locations *M* and *N*, respectively. $h_{1}$ and $h_{2}$ are the fitted plane elevation at locations *M* and *N*. All fitted planes are parallel to the plane fitting the reference glacier surface.

Here let us take two footprints *M* and *N* on complex terrain acquired at different times (Figure 4), where (*E*_1_, *N*_1_) and (*E*_2_, *N*_2_) are the horizontal coordinates of footprints *M* and *N*, respectively. For convenience of notation, the first year glacier surface among multi-year glacier thickness change is indicated as reference glacier surface (Figure 4). *H*_1_ and *H*_2_ are the GLAS elevations of *M* and *N* in the years *t*_1_ and *t*_2_. *H*_1_ and *H*_2_ can be decomposed into the elevation of the fitted plane ($h_{1}$, $h_{2}$), difference of elevation between the reference glacier surface (the one determined using the SRTM DEM) and the fitted plane ($\delta h_{1}$, $\delta h_{2}$), difference of elevation between the reference glacier surface and the true glacier surface ($\delta h_{s1}$, $\delta h_{s2}$), respectively. Thus, the differential equation between *H*_1_ and *H*_2_, can be written as:

(6) $$H_{2}-H_{1}=\alpha \times \left(E_{2}-E_{1}\right)+\beta \times \left(N_{2}-N_{1}\right)+\delta h_{s2}-\delta h_{s1}+\delta h_{2}-\delta h_{1}$$

Combining Equations (3), (4), and (6), the glacier thickness change (*dh*/*dt*) can be expressed as:

(7) $$\frac{dh}{dt}=(\delta h_{s2}-\delta h_{s1}+\delta h_{2}-\delta h_{1})/\left(t_{2}-t_{1}\right)$$

where ($\delta h_{s2}-\delta h_{s1}$)/(*t*_2_ − *t*_1_) is glacier thickness change, ($\delta h_{2}-\delta h_{1})$/(*t*_2_ − *t*_1_) is an error term. As illustrated in Figure 4, if ($\delta h_{2}-\delta h_{1})$ is greater than zero and *t*_2_ is greater than *t*_1_, the glacier thickness change $\frac{dh}{dt}$ will be larger than true value, and vice versa. When the glacier thickness change $\frac{dh}{dt}$ is calculated by solving all the differential equations in Equation (5), whether $\frac{dh}{dt}$ is larger or smaller than the true value depends on whether the overall ($\delta h_{2}-\delta h_{1})$ is negative or positive. In general, when applying the planes fitted to footprints at different times, ($\delta h_{2}-\delta h_{1})$ will not be zero due to the deviation of the fitted planar facets from the real glacier surface, which will bring errors in the retrieved glacier thickness change.

### 3.2. Improved Method Applicable to Complex Terrain

As Section 3.1 shows, the deviation of the fitted planar facets from the real glacier surface will bring errors in the retrieved glacier thickness change. Thus, curve fitting with a binary polynomial function was used to replace planar fitting to solve this problem. Equations (1) and (2) can be then rewritten as:

(8) $$H_{1}=\overset{p}{\underset{j=1}{\sum }}\overset{j}{\underset{i=0}{\sum }}a_{ji}E_{1}^{i}N_{1}^{j-i}+h_{1}$$

(9) $$H_{2}=\overset{p}{\underset{j=1}{\sum }}\overset{j}{\underset{i=0}{\sum }}a_{ji}E_{2}^{i}N_{2}^{j-i}+h_{2}$$

where *p* is the order of the binary polynomial function and is an integer value greater than 0, $a_{ji}$ are the coefficients of the binary polynomial function. $E_{1}^{i}N_{1}^{j-i}$ and $E_{2}^{i}N_{2}^{j-i}$ are the term of binary polynomial function.

By differentiating each pair of surfaces, Equation (5) can be rewritten as:

(10) $$\left(\begin{array}{c} dH_{1}\\ dH_{2}\\ \vdots \\ dH_{n} \end{array}\right)=\left(\begin{array}{c} dE_{1}^{p}\\ dE_{2}^{p}\\ \vdots \\ dE_{n}^{2} \end{array}\;\begin{array}{c} dN_{1}^{p}\\ dN_{2}^{p}\\ \vdots \\ dN_{n}^{2} \end{array}\;\begin{array}{c} \ldots \\ \ldots \\ \ddots \\ \ldots \end{array}\;\begin{array}{c} \Delta t_{1}\\ \Delta t_{2}\\ \vdots \\ \Delta t_{n} \end{array}\right)\cdot \left(\begin{array}{c} a_{p0}\\ a_{0p}\\ \vdots \\ \frac{dh}{dt} \end{array}\right)$$

where $a_{p0}$ and $a_{0p}$ are the coefficients of the binary polynomial function, subscript *n* is the number of differential equations. Glacier thickness change $\frac{dh}{dt}$ and the $a_{p0}$ and $a_{0p}$ coefficients can be obtained by solving Equation (10). Here, *p* is the order of the binary polynomial function, which indicates the complexity of the system of differential equations (Equation (10)). The greater the value of *p*, the more GLAS data are needed to solve Equation (10). If *p* is as small as 1 (i.e., planar fitting), then the calculated glacier thickness change may be affected by the fitting error as shown in Equation (7). Thus, it is important to choose a suitable *p* for capturing better the glacier surface, while keeping the order of the matrix differential equation as small as possible. The reference glacier surface is determined using the 2000 SRTM DEM that has spatially-continuous elevation data at a 90 m × 90 m spatial resolution.

### 3.3. Setting of Parameter p on the Basis of SRTM DEM

**Figure 5:**
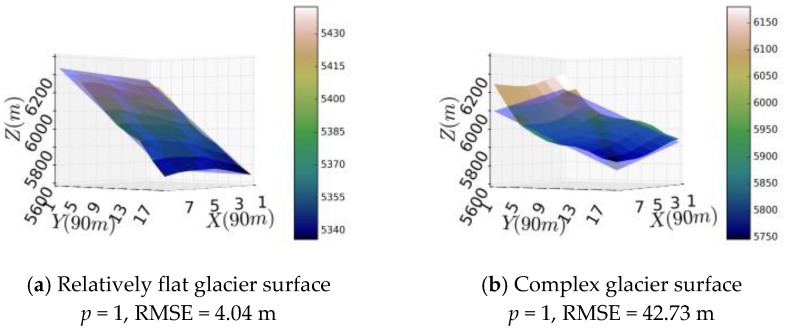

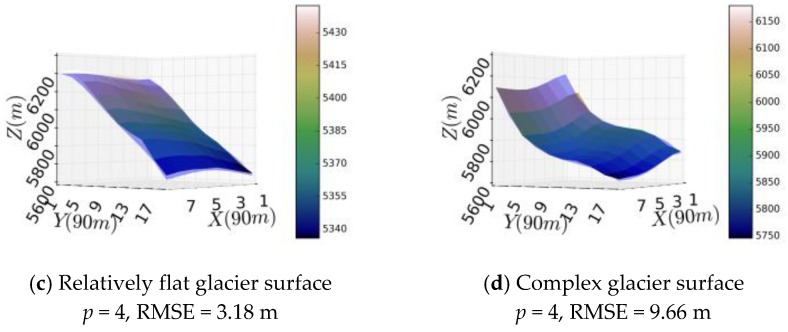
(**a**,**b**): the fitted surface and real surface when *p* = 1; (**c**,**d**): the fitted surface and real surface when *p* = 4. The light blue surface represents the fitted surface and the gradient colored surface represents the real surface.

In order to determine a suitable value of *p*, a binary polynomial function was used to fit to a relatively flat glacier surface and a relatively complex glacier surface based on SRTM elevations at the locations (84.45° *E*, 28.27° *N*) and (86.41° *E*, 28.30° *N*) of the Himalayas, respectively. After the tests on *p* values from 1 to 5, we found that the binary polynomial function with *p* = 4 could fit both the relatively flat glacier surface and the complex glacier surface and the root-mean-square-error (RMSE) value for *p* = 4 is much smaller than the RMSE value for *p* = 1, especially on relatively complex glacier surfaces (Figure 5). To better show the performance of polynomial fitting (*p* = 4), six more complex glacial facets were fitted by planar (*p* = 1) and polynomial functions (*p* = 4). The results show that the RMSE of polynomial fitting were rather small, while the RMSE of the planar fitting were much larger (Table 2). 

## 4. Results

### Application of the Improved Method to the Four Glaciers

The improved method using polynomial fitting was applied to the four test sites on the Naimona’nyi glacier, the Yanong glacier, the Chasku Muba glacier, and the Guliya glacier, respectively. In each test site, the facet width sampled by GLAS footprints equals the inter-track distance (Figure 2). For better measurement of the actual change in glacier surface elevation, and to reduce the influences of low GLAS data density, SRTM DEM grid values were used as measurements in the year 2000 and each grid was taken as one footprint. Additionally, in order to demonstrate the capability of the improved method to give more accurate estimates of glacier thickness change, the method of fitting a linear trend was used to calculate glacier thickness changes for Guliya and Chasku Muba glacier test sites. This method fits a multi-year linear trend to glacier elevation differences with respect to a reference DEM [8,9,10].

For Naimona’nyi glacier and Yanong glacier, by stretching or shortening the length of the along-track facet in one direction, four different lengths of the facet along track were used to test the influence of facet size on the results with the length increasing from 1000 m to 2500 m in 500 m increments (Table 3). The results of the glacier trend obtained with the planar fitting method (equivalent to the improved method with *p* = 1) showed large difference ranging from −0.55 m/year to −3.28 m/year for Naimona’nyi glacier and from 2.6 m/year to 4.39 m/year for Yanong glacier, respectively, when changing the length of the glacier facets from 1000m to 2500 m (Table 3). On the contrary, the results of the glacier trend obtained with the improved method (*p* = 4) showed small difference with values from −0.46 m/year to −0.97 m/year (Naimona’nyi glacier) and −0.45 m/year to −1.07 m/year (Yanong glacier), respectively, when glacier facets changing from 1000m to 2500 m. Furthermore, in the upper parts of Naimona’nyi glacier, Tian [24] and Zhu [25] measured elevation changes with a differential global positioning system (DGPS) and the results showed that the mean glacier thickness change from 2008 to 2010 and from 2005 to 2013 was about −0.67 m/year and −0.45 m/year, respectively. As the two results calculated by the improved method and measured by DGPS represent different periods, it is difficult to compare these two results with each other. The results are relatively close to each other, however, indicating that our method may be applicable to estimate changes in glacier thickness on large mountain glaciers. On the contrary, the results with the planar fitting method were close to the ones with the improved method with window length = 1000 m. With an increasing window lengths, the results obtained with the planar fitting method gave much large negative thickness change trends compared with the measured value −0.45 m/year in [24] and −0.67 m/year in [25] for the Naimona’nyi glacier (Table 3). For the Yanong glacier, although there is no measured glacier thickness change, Nie [26] and Liu [27] estimated the glacier retreat of 73 m/year from 1981 to 2001 based on Landsat data, which indicates that the positive glacier thickness change calculated by the planar fitting method may have a large bias. The large variation with glacier facet length and the very different estimated glacier thickness change could be caused by the planar fitting error, as the glacier surface have different complexity with increasing facet lengths.

For the Chasku Muba glacier as complex topography case and the Guliya glacier as large area case, the glacier thickness change results were also compared with the estimates by fitting a linear temporal trend. For the Chasku Muba glacier, the result obtained with the polynomial fitting method was 0.83 m/year, consistent with the result obtained with the linear temporal trend and much more reasonable than the 4.39 m/year value obtained with the planar fitting method. For the Guliya glacier, the glacier thickness change was estimated for six windows sizes along the ICESat track corresponding to six facets *W_1_ … W_6_*. The results obtained with the planar fitting method (Table 4) are not reliable as shown by the values for the facets *W_1_ … W_6_*. Moreover, differences across samples facets are large and the ones for facets W_4_ and W_5_ are inconsistent with the results obtained with polynomial fitting and with the linear temporal trend method. The results obtained with the polynomial fitting method decrease slightly from W_1_ to W_6_ and are pretty consistent with the result obtained by fitting a linear temporal trend, except for W_1_ and W_6_ where the fitting of a linear temporal trend have a low confidence (q = 0.32 and 0.86). 

## 5. Discussion

### 5.1. Discussion on the Assumptions

In the Introduction section, we stated that two assumptions were necessary for the planar fitting method. To represent better the local shape of a glacier surface, see Assumption 1, a binary polynomial function was used in the improved method. Assumption 2 may be difficult to hold true, especially when an along-track facet samples both the accumulation and the ablation area. We designed a set of experiments on the Himalayas glaciers to check whether the facet shape on the glacier remains unchanged with time. In each experiment, one year of GLAS data and SRTM-DEM within the facet were used to calculate the coefficients of the binary polynomial function by Equation (10) and *p* = 4 to show the shape of glacier surface in the year that the GLAS data was acquired. Besides, all years of GLAS data and SRTM data within the facet were also used to calculate the coefficients of the binary polynomial function. As the coefficients reflect the shape of glacier surface, the coefficients of each experiments were compared with each other to show the changes of shape of glacier surface with different years. The results show that the value of coefficients in different experiments is generally similar but has a large change in the *N* direction for the cases of *N^4^*, *N^3^*, and *N^2^* (Table 5). At the same time, the maximum distance along *N* and *E* direction sampled by one year of GLAS data within the facet reached about 1.5 km and 0.5 km respectively, which indicates that the shape of the facet changed in different years. Therefore, the estimated glacier surface is actually the mean surface across multiple years and the estimated *dh*/*dt* is the mean glacial thickness change.

### 5.2. The Impact of a Non-Rigid Facet on the Calculated Mean Glacier Thickness Change

As elaborated in Section 5.1, the glacier surface may not be exactly rigid, which will increase the uncertainty of estimated changes in glacier surface elevation especially when the GLAS data did not fully sample the glacier facets. Here, in order to quantify the uncertainty due to the fluctuation of glacial thickness change estimated by sampling different parts of glacier facets, we randomly chose 70% of GLAS data to estimate glacier thickness change on the Naimona’nyi and Yanong glaciers with the facet length of 1.5 km, the Chasku Muba glacier with facet length of 1 km. This procedure was replicated 50 times by randomly selecting the 70% of GLAS data each time so that every footprint in the facets could be used. Both methods with *p* = 1 and *p* = 4 were tested. Each calculation used 70% of GLAS data, i.e., by sampling part of the facet to yield the estimated glacier thickness change. The standard deviation over the 50 iterations provides information on the spatial variability of glacier thickness change. Table 6 gives the mean value and three times the standard deviation σ for the three test sites. The results show that the mean value is consistent with the value in Table 3 and Table 4 estimated by all the GLAS data and that the σ value of each estimate is relatively small, indicating that the change of shape within the facets is small. Additionally, the σ values of glacier thickness change estimated by the polynomial fitting method are smaller than the values from the planar fitting method, indicating that the polynomial fitting method is less affected by non-rigid facets than the planar fitting method.

### 5.3. Shortcomings of the Polynomial Fitting Method

**Figure 6:**
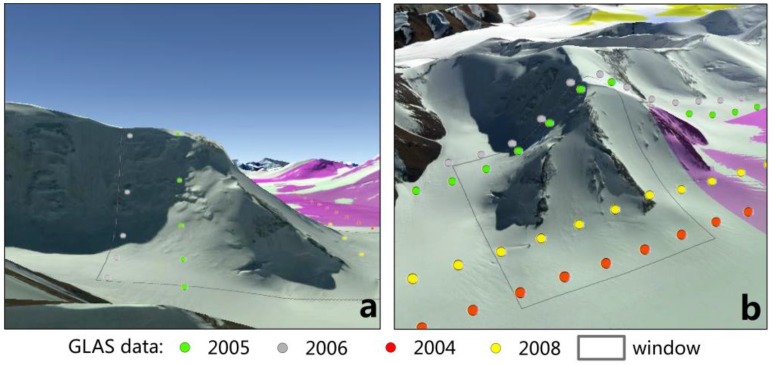
Demonstration of glacial surface with extreme complex topography. Background images are from Google Earth. Footprints is GLAS data. (**a**): Lateral view; and (**b**): front view.

The experiments above showed the capability of the polynomial fitting method to represent both a complex glacier surface and a large glacier surface. However, it is hard to apply it to a glacier surface with extremely complex topography. As shown in Figure 6 a glacier at (77.16° *E*, 35.91° *N*) from Karakoram Mountains, the topography changes abruptly within the selected facet. Planar, polynomial, and linear temporal trend fitting methods were applied to the GLAS footprints sampling the glacier surface (the window shown in Figure 6) from 2004 to 2008. The glacier thickness changes from the polynomial fitting method, the planar fitting method and the linear temporal trend method are −1.5 m/year, −2.94 m/year and 0.18 m/year, respectively, with low confidence (Table 7). For this specific glacier facet shown in Figure 6, the polynomial fitting method could not capture this complex glacier surface, a challenge augmented by the steeper slope. 

## 6. Conclusions

This study analyzed the error sources of the planar fitting method when applied to a complex glacier surface and found that the deviation between the fitted plane and the real glacier surface could cause a bias on the estimated glacier thickness change. Based on the analysis of error sources, we proposed to apply curve fitting by a binary polynomial function instead of planar fitting. Two groups of experiments based on SRTM DEM elevation data of different glacier surfaces were designed to determine a satisfactory order of the binary polynomial function, we concluded that a fourth-order binary polynomial function was appropriate. Considering that GLAS footprints are sparsely distributed on mid-low latitude glaciers, the SRTM DEM was used for the evaluation, which may be affected by errors of the SRTM DEM elevation data. It was more important, however, to have denser and regularly-spaced elevation data to evaluate the planar and polynomial fitting accuracy. The results for the two test sites on Yanong and Naimona’nyi glaciers also showed that polynomial fitting gives more reliable estimates than the planar fitting method. Furthermore, the results with the polynomial fitting for the Naimona’nyi glacier agree well with the in situ measurements, indicating that the polynomial fitting performs better than the planar fitting. The results for the two test sites on the Chasku Muba glacier and Guliya glacier showed that the polynomial fitting could deal with larger glaciers and glaciers with complex topography. Additionally, the polynomial fitting appears to have a good potential towards retrieving glacial thickness change on larger mountain glaciers only using ICESat-2 and without the need of extra DEMs, with the former to be launched in 2018 and have much denser footprints.

## Figures and Tables

**Table 1:** Area, roughness, and number of footprints acquired in the four glaciers. “-“ : No GLAS data.

	2003	2004	2005	2006	2007	2008	2009	Area (km^2^)	Roughness (m)
Naimona’nyi Glacier	-	3	9	11	8	-	19	7.3	10.6
Yanong Glacier	-	13	24	-	29	21	33	17.9	49.1
Guliya Glacier	-	79	61	3	53	59	6	111.3	14.7
Chasku Muba Glacier	-	39	35	32	-	37	-	43.7	38.4

**Table 2:** The RMSE value (m) between fitted glacial surface and real glacial surface for the cases of polynomial fitting (*p* = 4) and of planar fitting (*p* = 1) over six glacial facets in the Himalayas.

Polynomial Fitting (*p* = 4)	Planar Fitting (*p* = 1)	Longitude	Latitude
12.79	54.68	90.886	33.532
12.55	72.15	90.872	33.53
4.74	21.67	82.327	34.757
12.27	29.51	82.377	34.775
12.59	35.91	77.464	35.733
5.34	22.9	77.639	35.688

**Table 3:** Glacier elevation trends on Naimona’nyi glacier and Yanong glacier, respectively, with window lengths from 1000 m to 2500 m. Length: rectangle length along track.

*dh*/*dt* (m/year)	Polynomial Fitting (2000–2008/2009)				DGPS Measurement [25] (2008–2010)	DGPS Measurement [24] (2005–2013)
		*p*	1	4		
	Length					
Naimona’nyi Glacier	1000 (m)		−0.55	−0.66	−0.67	−0.45
	1500 (m)		−1.88	−0.46		
	2000 (m)		−2.45	−0.97		
	2500 (m)		−3.28	−0.82		
Yanong Glacier	1000 (m)		2.89	−1.07	*	*
	1500 (m)		4.39	−0.84		
	2000 (m)		2.82	−0.78		
	2500 (m)		2.6	−0.45		

*: No measured value.

**Table 4:** Glacier elevation trends on Chasku Muba glacier with complex topography and Guliya glacier with a large area. q: probability value, the lower the value, the higher the credibility; W*_i_*: different segments on Guliya glacier illustrated in Figure 2.

*dh*/*dt* (m/year)	Polynomial Fitting (2000–2008/2009)			Linear Temporal Trend (2004–2008/2009)	
	*P=*	1	4	*dh*/*dt*	q
Guliya glacier	W_1_	1.64	1.06	0.39 ± 0.78	0.32
	W_2_	0.64	0.60	0.67 ± 0.54	0.01
	W_3_	0.74	0.39	0.48 ± 1.3	0.4
	W_4_	3.6	0.47	0.61 ± 0.8	0.03
	W_5_	−0.94	0.35	0.42 ± 0.58	0.09
	W_6_	0.32	0.39	0.04 ± 0.52	0.86
Chasku Muba Glacier		4.39	0.83	0.58 ± 0.71	0.11

**Table 5:** Coefficients of fitted glacier surface using different year of GLAS data and SRTM DEM. *E, N* are the variables of polynomial function and the different combination of E, N are the different terms of polynomial function (Equation (8)).

Year of GLAS	*E^4^*	*N^4^*	*E^3^N*	*E^2^N^2^*	*EN^3^*	*E^3^*	*N^3^*	*E^2^N*	*EN^2^*	*E^2^*	*N^2^*	*EN*	*E*	*N*
2004	–1.07 × 10^4^	**5.07 × 10^7^**	1.17 × 10^5^	–2.39 × 10^6^	4.19 × 10^7^	–6.22 × 10^0^	**–2.88 × 10^3^**	6.43 × 10^1^	–1.14 × 10^3^	–3.14 × 10^–3^	**8.91 × 10^–2^**	3.31 × 10^–2^	–2.36 × 10^–1^	2.04 × 10^–1^
2006	–1.09 × 10^4^	**–1.75 × 10^8^**	1.20 × 10^5^	–2.51 × 10^6^	4.67 × 10^7^	–6.18 × 10^0^	**6.33 × 10^3^**	6.89 × 10^1^	–1.29 × 10^3^	–3.13 × 10^–3^	**–1.27 × 10^–1^**	3.61 × 10^–2^	–2.47 × 10^–1^	1.97 × 10^–1^
2007	–8.30 × 10^3^	**1.80 × 10^8^**	8.27 × 10^4^	–1.62 × 10^6^	2.61 × 10^7^	–4.82 × 10^0^	**–2.97 × 10^3^**	4.63 × 10^1^	–7.12 × 10^2^	–2.30 × 10^–3^	**5.64 × 10^–2^**	2.00 × 10^–2^	–2.49 × 10^–1^	1.96 × 10^–1^
2008	–1.11 × 10^4^	**–1.82 × 10^8^**	1.18 × 10^5^	–2.42 × 10^6^	4.36 × 10^7^	–6.36 × 10^0^	**4.45 × 10^3^**	6.81 × 10^1^	–1.26 × 10^3^	–3.26 × 10^–3^	**–1.58 × 10^–1^**	3.45 × 10^–2^	–2.48 × 10^–1^	2.05 × 10^–1^
all	–1.08 × 10^4^	**1.62 × 10^7^**	1.11 × 10^5^	–2.16 × 10^6^	3.83 × 10^7^	–6.20 × 10^0^	**2.67 × 10^3^**	6.20 × 10^1^	–1.06 × 10^3^	–3.12 × 10^–3^	**–8.13 × 10^–2^**	3.11 × 10^–2^	–2.34 × 10^–1^	2.03 × 10^–1^

**Table 6:** Statistics of the glacier thickness change estimation from the 50 duplications of randomly selecting 70% GLAS data for calculation.

*dh*/*dt* (m/year)	Planar Fitting (*p* = 1)		Polynomial Fitting (*p* = 4)	
	Mean	3 × σ	Mean	3 × σ
Naimona’nyi Glacier	−1.83	0.13	−0.46	0.08
Yanong Glacier	3.34	0.25	−0.77	0.15
Chasku Muba Glacier	4.44	0.81	0.85	0.45

Note: **σ**: standard deviation.

**Table 7:** Glacier thickness change trends on glacier surface with extremely complex topography as shown in Figure 6. *q*: probability value. The lower the value *q*, the higher the credibility.

Planar Fitting (*p* = 1)	Polynomial Fitting (*p* = 4)	Fitting a Trend (2004–2008)	
		*dh*/*dt*	*q*
−2.94	−1.5	−0.18 ± 4.18	0.93
